# Gut Microbiota, Microinflammation, Metabolic Profile, and Zonulin Concentration in Obese and Normal Weight Subjects

**DOI:** 10.1155/2013/674106

**Published:** 2013-07-18

**Authors:** Agnieszka Żak-Gołąb, Piotr Kocełak, Małgorzata Aptekorz, Maria Zientara, Łukasz Juszczyk, Gayane Martirosian, Jerzy Chudek, Magdalena Olszanecka-Glinianowicz

**Affiliations:** ^1^Pathophysiology Unit, Department of Pathophysiology, Medical University of Silesia, University of Silesia, 18 Medyków Street, 40-752 Katowice, Poland; ^2^Health Promotion and Obesity Management Unit, Department of Pathophysiology, Medical University of Silesia, University of Silesia, 18 Medyków Street, 40-752 Katowice, Poland; ^3^Department of Medical Microbiology, Medical University of Silesia, University of Silesia, 18 Medyków Street, 40-752 Katowice, Poland

## Abstract

The association between gut microbiota and circulating zonulin level, a marker of intestinal permeability, has not been studied yet. The aim of the study is the assessment of plasma zonulin, haptoglobin and proinflammatory cytokines (TNF-**α** and IL-6) levels in relation to composition of gut microbiota in obese and normal weight subjects. Circulating inflammation markers, such as TNF-**α**, sTNFR1, sTNFR2, IL-6, zonulin, and haptoglobin levels were measured and semiquantitative analysis of gut microbiota composition was carried out in 50 obese and 30 normal weight subjects without concomitant diseases. Higher circulating zonulin, TNF-**α**, sTNFR1, sTNFR2, and IL-6 levels were found in the obese subjects. Plasma zonulin level correlated positively with age (*r* = 0.43, *P* < 0.001), body mass (*r* = 0.30, *P* < 0.01), BMI (*r* = 0.33, *P* < 0.01), fat mass and fat percentage (*r* = 0.31, *P* < 0.01 and *r* = 0.23, *P* < 0.05, resp.). Positive correlations between bacterial colony count and sTNFR1 (*r* = 0.33, *P* < 0.01) and plasma zonulin (*r* = 0.26, *P* < 0.05) but not haptoglobin levels were found. Additionally, plasma zonulin level was proportional to daily energy intake (*r* = 0.27, *P* < 0.05) and serum glucose concentration (*r* = 0.18, *P* < 0.05) and inversely proportional to diet protein percentage (*r* = −0.23, *P* < 0.05). Gut microbiota-related systemic microinflammation in the obese is reflected by circulating zonulin level, a potential marker of interstitial permeability.

## 1. Introduction

The results of numerous studies suggested that changes in the composition of gut microbiota are factors participating in the development of obesity by obtaining extra energy from the portion of food, reduced expression of FIAF (fasting-induced adipocyte factor) in the enterocytes with inhibitory activity on intestinal lipoprotein lipase, and the increased release of peptide YY that slows the intestinal motility [1].

Obesity, especially visceral, is associated with systemic microinflammation [2–4]. Adipocytes and even more macrophages infiltrating visceral adipose tissue in obese subjects are the source of circulating proinflammatory cytokines, such as TNF-*α* and IL-6 [5, 6]. However, in the recent years it was also suggested that alteration in gut microbiota composition followed by an impairment of intestinal wall integrity is additional factor escalating systemic microinflammation, at least in the obese [7]. Systemic microinflammation is an important link in the pathogenesis of insulin resistance and comorbidities related to obesity, such as hypertension, dyslipidemia, and type 2 diabetes [8, 9].

The discovery of zonula occludens toxin (Zot), a Vibrio cholera enterotoxin, and later its eukaryotic counterpart zonulin increased cognition of the mechanisms that regulate the intestinal paracellular pathway. Zonulin is a mediator known to regulate intestinal permeability by modulating intracellular tight junctions (TJs) [10, 11]. Human zonulin (47-kDa protein) increases intestinal permeability in small intestine and participates in the development of intestinal innate immunity [11], encoded by haptoglobin 2 gene [12]. Circulating zonulin is considered as a potential marker of intestinal permeability [13]. The results of recently published study revealed higher circulating zonulin level in obese than in nonobese subjects and in subjects with glucose intolerance in comparison to group with normal glucose tolerance. Moreover, the positive correlation between serum zonulin level and BMI, WHR, fasting insulin, and triglycerides levels as well as plasma IL-6 concentration but negative with HDL-cholesterol level and insulin sensitivity was found [14]. Therefore, we hypothesized that circulating zonulin levels may be a link between alteration in the gut microbiota composition and systemic microinflammation in obese subjects.

The aim of the study is the assessment of plasma zonulin, haptoglobin, and proinflammatory cytokines (TNF-*α* and IL-6) levels in relation to composition of gut microbiota in obese and normal weight subjects.

## 2. Materials and Methods

Eighty subjects without concomitant diseases, 50 obese (39 women and 11 men) and 30 normal weight (24 women and 6 men), were enrolled. Subjects with acute or chronic diseases, any drug use, including antibiotics and oral contraceptive agents, body mass change exceeding more than 3 kg during preceding 6 months, cigarette smoking, drinking more than 3 drinks per week, endocrine disorders, that is hyper- and hypothyroidisms, Cushing's syndrome, and polycystic ovary syndrome were excluded. The characteristics of study group are presented in Table 1.

The study protocol was approved by the Bioethics Committee of Medical University of Silesia (KNW/0022/KB1/41/10). Informed consent was obtained from each study participant.

Anthropometric parameters (body mass, height, and waist circumference) were measured in the morning between 8 and 9 after 16-hour overnight fast. BMI was calculated according to standard formula. Body composition was measured using the bioimpedance method (Bodystat 1500, Douglas, Isle of Man).

Dietary energy and macronutrients intake were assessed on the basis of a three-day food diary completed by each study subject. The computer database of foods from the National Food and Nutrition Institute (Diet 4.0, Polish Food Tables 2005) was used to calculate the energy and micronutrient dietary intake.

### 2.1. Biochemical Measurements

The 8 mL samples of venous blood were collected in the morning between 8 and 9 a.m., after overnight fast (16 h), according to the recommendations of the kit manufacturers. Serum and plasma samples were stored at −80°C. Serum glucose, total cholesterol, LDL, and HDL cholesterol as well as triglycerides were estimated by colorimetric methods using a commercially available test kit (Roche Diagnostics GmbH, Mannheim, Germany). Serum insulin concentration was determined by electrochemiluminescence method (Cobas e411, Roche Diagnostics GmbH, Mannheim, Germany) with a lower limit of sensitivity of 1.2 *μ*IU/mL and intra- and inter-assay coefficients of variations of 5.2% and 5.8%, respectively. HOMA-IR was calculated using the standard formula: HOMA-IR = fasting concentration of insulin (*μ*IU/mL) × fasting concentration of glucose (mmol/L)/22.5. Insulin resistance was diagnosed with HOMA-IR value above 2.49.

The plasma concentrations of TNF-*α*, sTNFRs, and IL-6 were measured using a commercially available highly sensitive ELISA kits (R&D Systems, MN, U.S.A). The sensitivity of the TNF-*α* assay was less than 0.1 pg/mL. Mean intra-assay coefficient of variance was less than 4.3% and mean inter-assay coefficient of variance was 7.3%. The sensitivities of the sTNFR1 and sTNFR2 assays were typically less than 0.77 pg/mL and 0.6 pg/mL, respectively. Mean intra-assay coefficients of variance were <3.6% and 2.6%, respectively, and mean inter-assay coefficients of variance were 3.7% and 3.5%, respectively. The sensitivity of the IL-6 assay was less than 0.04 pg/mL. Mean intra-assay coefficient of variance was less than 7.8% and mean inter-assay coefficient of variance was 7.2%.

Plasma zonulin concentrations were measured by ELISA (Immundiagnostik AG, Bensheim, Germany). The sensitivity of the assay was less than 0.01 ng/mL. Mean intra- and inter-assay coefficients of variance were 5% and 8.5%, respectively. The K5600 ELISA kit used for zonulin measurement detects only the active form of zonulin.

Plasma haptoglobin concentration was assessed by ELISA (AssayPro, Saint Charles, MO, U.S.A). The sensitivity of the assay was less than 0.07 *μ*g/mL. Intra-assay and inter-assay coefficients of variation were 4.9% and 7.5%, respectively.

### 2.2. Analysis of Fecal Microflora

**Figure 1:**
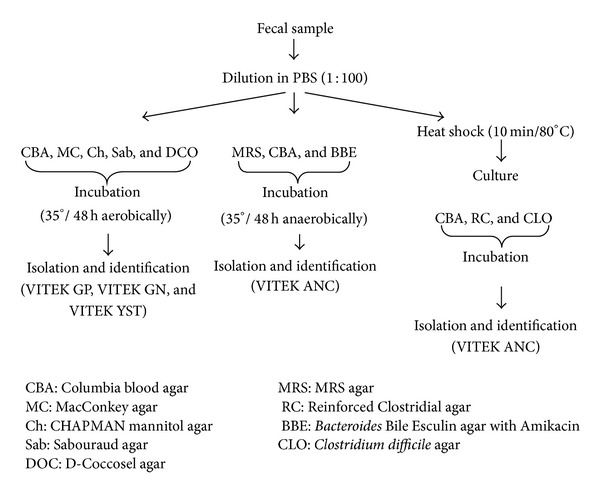
Fecal samples processing scheme.

All 80 fecal samples were obtained from study subjects without use of laxatives, preventing contamination with urine. The samples were collected in a sterile disposable container without fluid and delivered to the laboratory within two hours on the day of blood sample collection and processed according to the scheme presented in Figure 1. Each fecal sample was diluted (1 : 100) in PBS, cultured using the appropriate media (numbers 1–9) in aerobic (numbers 1–5) and anaerobic (numbers 6–9) conditions, respectively:

1. CBA: Columbia blood agar × 2 (bioMerieux, Marcy L'Etoile, France),
2. MC: Mac Conkeyagar (Becton, Dickinson and Company, France),
3. Ch: CHAPMAN agar with mannitol (Becton, Dickinson and Company, USA),
4. Sab: Sabouraud agar (bioMerieux, Marcy L'Etoile, France),
5. DCO: D-Coccosel agar (bioMerieux, Marcy L'Etoile, France),
6. MRS: MRS agar (Becton, Dickinson and Company, France),
7. RC: Reinforced Clostridial agar (Oxiod, UK),
8. BBE: *Bacteroides* Bile Esculin agar with Amikacin (BD BBL, Germany),
9. CLO: *Clostridium difficile* agar (bioMerieux, Marcy L'Etoile, France).

About 1 g of each fecal sample was subjected to 10 min of heat shock (800C, Termoblock RED-HOT 35) and cultured onto Columbia blood (number 1) and Reinforced Clostridial (number 7) agars for 3–5 days in anaerobic conditions (Whitley A-35 Anaerobic Workstation, UK). After incubation all plates were evaluated and bacterial colonies were encountered, Gram-stained, and identified using appropriate cards (GP, GN, YST, and ANC) for automatic identification system of microorganisms—VITEK 2 compact (bioMerieux, Marcy L'Etoile, France). As reference strains of *Bacteroides ovatus* ATCC BAA-1296, *Clostridium septicum* ATCC 12464, *Clostridium perfringens* ATCC 13124, *Staphylococcus aureus* ATCC 25923, and *Escherichia coli* ATCC 25922 from ATCC collection were used.

CFU—colony forming unit—was defined as the number of growing colonies. The number was recalculated according to the dilution factor used.

### 2.3. Statistical Analysis

Statistical analysis was performed using the STATISTICA 10.0 PL software (StatSoft Poland, Cracow, Poland). The results are presented as median values with interquartile ranges. The Chi-square test was used for comparison of the frequency of qualitative variables in studied groups and Mann-Whitney *U* tests were used for comparison of quantitative variables between groups. The univariate correlation coefficients were calculated according to Spearman. Five models of multiple regression analyses for zonulin level and alternative sets of potentially explanatory variables were used: the total bacterial count and total *Bacteroides* and *Firmicutes* counts, energy intake and macronutrients content, and parameters of carbohydrate and lipid metabolism, as well as inflammation parameters. The results were considered as statistically significant with a *P* value of less than 0.05. 

## 3. Results

### 3.1. Characteristics of Study Groups

As a consequence of the inclusion criteria, body mass, BMI, and the body fat mass and percentage were higher in the obese than in normal weight group (Table 1). The obese group were older by 9 years in average than normal weight group.

Serum glucose, insulin, and triglycerides concentrations and HOMA-IR and Hb_A1c_ values were higher, while serum HDL-cholesterol level was significantly lower in obese group (Table 1). 

The mean daily energy consumption and the percentages of fat content in the diet were higher, while the percentage of carbohydrate and protein were lower in obese group than in normal weight group. The fiber consumption per daily energy intake was similar in both groups (Table 1). 

The total bacterial as well as *Bacteroides and Firmicutes* counts and the rate of *Bacteroides to Firmicutes* spp. were similar in both study groups (Table 1).

**Figure 2:**
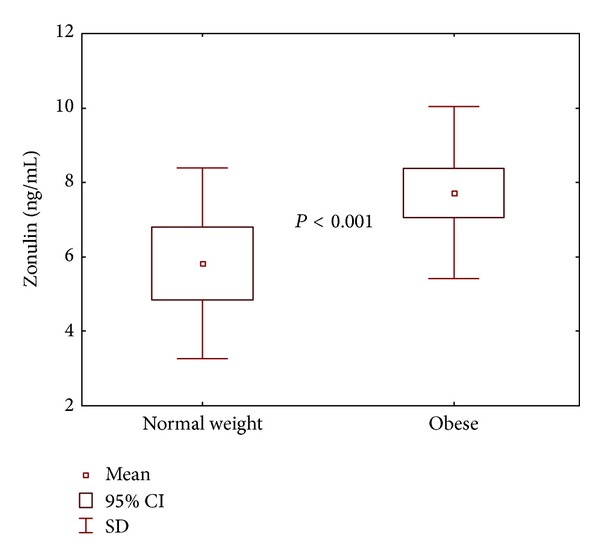
The comparison of mean zonulin concentrations in study groups.

Significantly higher plasma TNF-*α*, sTNFR1, sTNFR2, IL-6, and zonulin levels were observed in the obese group (Table 2, Figure 2), while plasma haptoglobin level did not differ statistically in both study groups. 

### 3.2. Factors Associated with Plasma Concentration of Zonulin

The whole study group was divided on the basis of the reference or median value of parameters of a total energy and macronutrients dietary intake, HOMA-IR value, intestinal bacterial counts, mediators of inflammation, glucose, and lipid profile (Table 3). In subgroups with sTNFR1 ≥ 1510 pg/mL, haptoglobin ≥ 1.42 *μ*g/mL, and daily energy intake ≥ 2095 kcal/d, significantly higher plasma zonulin levels were found.

### 3.3. Correlation Analyses

**Figure 3:**
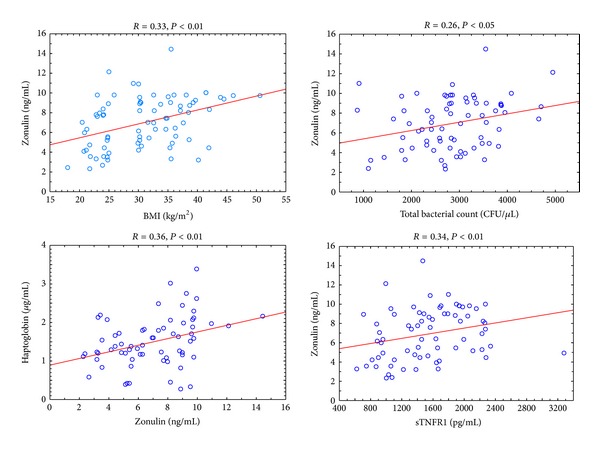
The correlations of zonulin and BMI, total bacterial count, haptoglobin, and sTNFR1 in all study subjects.

The correlation coefficients were calculated for all study subjects. Plasma zonulin level correlated positively with age (*R* = 0.43, *P* < 0.001), body mass (*R* = 0.30, *P* < 0.01), BMI (*r* = 0.33, *P* < 0.01) (Figure 3), fat mass and percentage (*R* = 0.31, *P* < 0.01 and *R* = 0.23, *P* < 0.05, resp.). Moreover, positive correlation between plasma zonulin level and total bacterial count (*R* = 0.26, *P* < 0.05) as well as sTNFR1 (*R* = 0.34, *P* < 0.01) and haptoglobin levels (*R* = 0.36, *P* < 0.01) (Figure 3) was shown. Furthermore, plasma zonulin was proportional to daily energy intake (*R* = 0.27, *P* < 0.05) and inversely proportional to protein percentage dietary intake (*R* = −0.23, *P* < 0.05). Additionally, serum glucose concentration correlated positively with zonulin level (Table 4).

Plasma haptoglobin correlated significantly only with age (*R* = 0.25, *P* < 0.05), fat mass and percentage (*R* = 0.26, *P* < 0.05 and *R* = 0.34, *P* < 0.01, resp.). 

Plasma sTNFR1 level also correlated significantly with the total bacterial count (*R* = 0.33, *P* < 0.01). Moreover, both plasma sTNFR1 and sTNFR2 as well as IL-6 levels were proportional to daily energy intake (*r* = 0.30, *P* < 0.01; *r* = 0.27, *P* < 0.05; and *r* = 0.31, *P* < 0.01, resp.). 

Neither *Bacteroides* and *Firmicutes* counts nor percentages correlated significantly with levels of zonulin and other inflammatory markers assessed.

Insulin levels and HOMA-IR correlated positively with circulating sTNFRs and IL-6 but not with TNF-*α* levels. Moreover, there was an inverse relation between concentrations of HDL-cholesterol and plasma TNF-*α*, sTNFR1, and IL-6 but not zonulin and sTNFR2 levels (Table 4). 

### 3.4. Multiple Regression Analyses

In multiple regression model including the composition of gut microbiota as an independent variable, zonulin level was only related to total bacteria count (*β* = 0.33 ± 0.13) but not to the count of *Bacteroides* or *Firmicutes*. Both BMI (*β* = 0.26 ± 0.10) and age (*β* = 0.31  ±  0.06) were in addition to total bacterial count (*β* = 0.23 ± 0.10) explanatory variables for circulating zonulin concentration. 

In the model including dietary variables, as independent variables, zonulin level was related to fat percentage in diet (*β* = 0.23 ± 0.11) and fiber intake in relation to daily energy consumption (*β* = 0.32 ± 0.12).

In the model including parameters of carbohydrates metabolism, zonulin variability was explained only by glucose levels (*β* = 0.38 ± 0.12). Zonulin variability was not related to lipid metabolism parameters. 

## 4. Discussion

In accordance with recently published study [14], we observed higher circulating zonulin level, a known mediator of intestinal permeability, modulating intracellular tight junctions (TJs) [9, 10] in obese subjects. Additionally, we observed that plasma zonulin, but not haptoglobin level, was proportional to total bacteria count.

Recently a lot of interest has raised the role of gut microbiota in the pathogenesis of obesity and its concomitant diseases [15]. Bacteria colonizing the gut are the source of shell fragment G(−) bacteria, lipopolysaccharide (LPS), an inducer of low-grade chronic inflammation. Increased concentrations of LPS in the gut and in the plasma were observed in obese, diabetic subjects consumed a rich-fat diet [16, 17]. LPS stimulates secretion of proinflammatory cytokines, such as TNF-*α*, IL-1, and IL-6 by immune cells [15]. 

It should be emphasized that in our study neither *Bacteroides* and *Firmicutes* spp. counts nor their percentages were associated with the levels of circulating zonulin and other assessed inflammatory markers. It is suggested that for the induction of low-grade inflammation in the obese, total bacteria count is more important than the gut microbiota composition. Abundant gut microbiota and its composition in the obese depend on the energy consumption and diet composition [18]. 

The higher daily energy consumption in the obese is another explanation for systemic microinflammation expressed by increased circulating zonulin levels. We observed that zonulin level was associated with daily energy consumption in a univariate analysis only with diet composition (fiber intake in relation to daily energy consumption and fat percentage in diet) in a multiple regression analysis; thus circulating plasma zonulin concentration is mostly associated with higher fat consumption causing the increased daily energy intake. It seems that fat consumption may stimulate bacteria growth, while fiber is the substrate for fermentation in the colon. The process of gut microbiota-dependent fermentation of indigestible polysaccharides in the colon is the source of gaining extra energy from food. The fermentation product, propionic acid, is a substrate for the gluconeogenesis and lipogenesis [19].

Moreno-Navarrete et al. [14] have shown higher circulating zonulin level in subjects with impaired than normal glucose tolerance irrespective of body mass. Thus, zonulin seems to be one of factors contributing to insulin resistance development; however, the association disappeared after adding plasma IL-6 level to the multiple regression analysis model [14]. We did not observe association between zonulin level and insulin resistance (scored as HOMA-IR above 2.49). This difference may be the result of remarkably lower percentage of insulin-resistant subjects in our study group. However, in accordance with the results obtained by Moreno-Navarrete et al. [14], relationship between circulating glucose and zonulin levels was shown in our study. 

In a similar way we failed to prove the association between serum lipid and circulating zonulin levels, previously described by Moreno-Navarrete et al. [14]. It should be emphasized that increased total cholesterol, LDL-cholesterol, and triglycerides as well as decreased HDL-cholesterol levels in our study group were less frequent than in the cited study.

We observed positive association between circulating zonulin and sTNFR1 levels. Additionally, plasma zonulin level was higher in the subgroups with sTNFR1 concentration above 1510 pg/mL (median value). The sTNFR1 is a sensitive marker of low-grade inflammation in the obese [20]. Thus, these results confirm that circulating zonulin is an inflammatory marker, as its precursor, haptoglobin (Hp) [12], a liver acute-phase response protein. The expression of Hp in hepatocytes is increased by a number of proinflammatory cytokines including IL-1, IL-6, and TNF-*α* [21, 22]. Our results are extending these findings, demonstrating the proportional relation between circulating levels of haptoglobin and zonulin. However, as both proteins are the products of the same gene, a stronger than shown (*R* = 0.36) correlation might be expected. This may suggest the different and perhaps organ-specific secretion of these proteins. Whether this hypothesis is true remains to be verified. Chiellini et al. showed that Hp expression is upregulated in the white adipose tissue (WAT) in the obese rodents depending on the TNF-*α* pathway [23]. It was also observed that the factor stimulating haptoglobin mRNA expression in adipose tissue to the levels comparable to those in liver is LPS. However, this stimulation is secondary to the enhanced release of proinflammatory cytokines such as IL-1, IL-6 and, TNF-*α* [22]. 

The results of numerous studies revealed that circulating Hp level is proportional to BMI and components of metabolic syndrome [23–26]. Contrary to Moreno-Navarrete et al. [14], we showed the positive correlation between BMI and zonulin level. These data suggest that zonulin, the product of the different splicing of Hp2 gene exons [20], is a new potential marker of systemic microinflammation associated with obesity and is more sensitive than haptoglobin [23, 27, 28]. However, the correlation between total bacteria count in feces and circulating zonulin in our study suggests that it is rather the maker of gut mucosa inflammation in the obese than in the visceral adipose tissue. Our hypothesis may be supported by some recent findings. It was shown that probiotics administration in patients diagnosed with colorectal carcinoma reduces postoperative septicemia and is associated with reduced circulating zonulin level [29]. Additionally, increased zonulin levels were found in septic patients, potentially reflecting increased intestinal permeability in sepsis [30]. As already mentioned, abundant growth of gut microbiota is the consequence of high energy consumption by the obese related to high dietary fat intake. Thus increased intestinal permeability in the obese may be the effect of long-lasting inappropriate nutritional habits. Further studies are necessary to confirm our hypothesis.

The limitation of our study, beyond the size of the study groups, is the methodology of microbiome analysis and the lack of endotoxin assessment as well as the assessment of energy and macronutrients dietary intake based on a three-day food diary only.

## 5. Conclusions

Gut microbiota-related systemic microinflammation in the obese is reflected by circulating zonulin level, a potential marker of interstitial permeability.

## Figures and Tables

**Table 1:** The comparison of anthropometric and metabolic parameters in the study groups (median values and interquartile ranges).

	Obese *N* = 50	Normal weight *N* = 30	Statistical significance
Age (years)	53.5 (42.0–63.0)	42.5 (32.0–52.0)	*P* < 0.01
BMI (kg/m^2^)	35.4 (30.6–38.7)	23.7 (21.8–24.8)	*P* < 0.001
Fat mass (kg)	43.3 (36.2–50.2)	20.1 (17.6–24.7)	*P* < 0.001
Fat (%)	46.7 (40.9–49.9)	32.5 (28.4–40.0)	*P* < 0.001
Total cholesterol (mg/dL)	183.0 (158–219)	195.0 (156–215)	Ns
LDL-cholesterol (mg/dL)	134 (103–172)	117 (94–140)	Ns
HDL-cholesterol (mg/dL)	46 (37–56)	58 (45–70)	*P* < 0.001
Triglycerides (mg/dL)	120 (92–156)	87 (66–109)	*P* < 0.001
Glucose (mmol/L)	4.9 (4.4–5.5)	4.6 (4.3–4.8)	*P* < 0.05
Insulin (*µ*U/mL)	11.1 (8.0–13.8)	7.4 (5.3–9.7)	*P* < 0.01
HOMA-IR	2.2 (1.7–3.2)	1.5 (1.1–1.9)	*P* < 0.01
Hemoglobin A1_C_ (%)	5.3 (4.8–5.6)	5.0 (4.8–5.3)	*P* < 0.05

Diet			
Energy intake (kcal/day)	2550 (2043–2923)	1625 (1488–1813)	*P* < 0.001
Protein (%)	14.0 (12.3–16.3)	16.3 (13.0–17.7)	*P* < 0.001
Fat (%)	43.0 (38.3–46)	37.7 (31.7–41.0)	*P* < 0.05
Carbohydrates (%)	43.7 (39.7–47.7)	46.7 (41.0–52.3)	*P* < 0.01
Fiber (g/1000 kcal)	8.0 (6.5–9.7)	6.1 (2.0–10.8)	Ns

Gut microbiota			
The total bacterial count (CFU/*µ*L)	3084 (2230–3571)	2725 (2422–2989)	Ns
*Bacteroides* (CFU/*µ*L)	800 (500–1000)	600 (500–812)	Ns
*Firmicutes *spp. (CFU/*µ*L)	532 (306–910)	716 (405–1000)	Ns
The rate of *Bacteroides*/*Firmicutes *spp.	1.1 (0.8–1.9)	0.7 (0.5–1.7)	Ns

**Table 2:** The comparison of inflammation parameters in the study groups (median values with interquartile ranges).

	Obese *N* = 50	Normal weight *N* = 30	Statistical significance
TNF-*α* (pg/mL)	1.5 (1.4–1.8)	1.3 (0.9–1.6)	*P* < 0.01
sTNFR1 (pg/mL)	1772 (1447–2079)	1049 (909–1486)	*P* < 0.001
sTNFR2 (pg/mL)	3414 (2717–3805)	2877 (2303–3244)	*P* < 0.01
IL-6 (pg/mL)	1.9 (1.8–2.5)	1.0 (0.8–2.1)	*P* < 0.001
Haptoglobin (*µ*g/mL)	1.5 (1.2–2.0)	1.3 (1.0–1.7)	Ns
Zonulin (ng/mL)	8.2 (7.1–8.4)	5.4 (4.8–6.8)	*P* < 0.001

**Table 3:** The comparison of zonulin concentrations in groups according to different parameters (mean and 95% confidential interval).

	Zonulin (ng/mL)	*P*
Gender		
Females; *n* = 54	7.1 (6.4–7.7)	
Males; *n* = 26	6.7 (5.5–8.0)	Ns

Diet		
Total energy intake < 2093 kcal/d; *n* = 40	6.3 (5.5–7.0)	
Total energy intake ≥ 2095 kcal/d; *n* = 40	7.8 (7.0–8.6)	*P* < 0.01
Fat < 41%; *n* = 41	7.2 (6.3–8.1)	
Fat ≥ 41%; *n* = 39	6.8 (6.1–7.6)	Ns
Carbohydrates < 44%; *n* = 41	6.9 (6.0–7.8)	
Carbohydrates ≥ 44%; *n* = 39	7.2 (6.4–7.9)	Ns
Fiber g/1000 kcal < 7.7; *n* = 67	7.0 (6.3–7.7)	
Fiber g/1000 kcal ≥ 7.7; *n* = 13	7.2 (3.9–10.4)	Ns

Lipid profile		
Total cholesterol < 200 mg/dL; *n* = 50	6.8 (6.1–7.5)	
Total cholesterol ≥ 200 mg/dL; *n* = 30	7.1 (6.1–8.1)	Ns
HDL < 40 (M) and <50 mg/dL (F); *n* = 37	7.2 (6.3–8.1)	
HDL ≥ 40 (M) and ≥50 mg/dL (F); *n* = 43	6.7 (5.9–7.5)	Ns
LDL < 135 mg/dL; *n* = 49	6.4 (5.6–7.1)	
LDL ≥ 135 mg/dL; *n* = 31	7.4 (6.5–8.2)	Ns
Triglycerides < 150 mg/dL; *n* = 60	7.3 (6.1–8.5)	
Triglycerides ≥ 150 mg/dL; 20	6.8 (6.1–7.5)	Ns

Insulin resistance		
Insulin level > 15.4 *µ*U/mL; *n* = 21	7.4 (6.3–8.4)	
Insulin level ≤ 15 *µ*U/mL; *n* = 59	6.6 (5.8–7.5)	Ns
HOMA ≥ 2.49; *n* = 28	7.6 (6.2–9.0)	
HOMA < 2.49; *n* = 52	6.5 (5.7–7.3)	Ns
Glucose ≥ 100 mg/dL; *n* = 12	8.2 (5.9–9.7)	
Glucose < 100 mg/dL; *n* = 68	7.0 (4.7–8.9)	Ns

Bacterial content		
Total bacterial count < 2829; *n* = 40	6.4 (5.6–7.3)	
Total bacterial count ≥ 2829; *n* = 40	7.5 (6.6–8.4)	Ns
*Bacteroides* count < 800; *n* = 39	6.6 (5.7–7.5)	
*Bacteroides* count ≥ 800; *n* = 41	7.4 (6.6–8.1)	Ns
*Firmicutes* count < 585, *n* = 39	6.9 (6.2–7.7)	
*Firmicutes* count ≥ 585; *n* = 41	7.1 (6.2–8.0)	Ns
*Bacteroides* < 26%; *n* = 38	7.1 (6.1–8.0)	
*Bacteroides* ≥ 26%; *n* = 42	6.7 (5.9–7.6)	Ns
*Firmicutes* < 22%; *n* = 40	7.3 (6.4–8.1)	
*Firmicutes* ≥ 22%; *n* = 40	6.6 (5.8–7.5)	Ns
*Firmicutes/Bacteroides* index < 1.1; *n* = 32	6.9 (5.9–7.8)	
*Firmicutes/Bacteroides* index ≥ 1.1; *n* = 48	7.1 (6.4–7.9)	Ns

Inflammatory markers		
TNF-*α* < 1.45 pg/mL; *n* = 38	7.2 (6.2–8.1)	
TNF-*α* ≥ 1.45 pg/mL; *n* = 42	6.7 (5.8–7.6)	Ns
sTNFR1 < 1510 pg/mL; *n* = 39	6.2 (5.2–7.2)	
sTNFR1 ≥ 1510 pg/mL; *n* = 41	7.6 (6.8–8.3)	*P* < 0.01
sTNFR2 < 3103 pg/mL; *n* = 40	6.5 (5.6–7.3)	
sTNFR2 ≥ 3103 pg/mL; *n* = 40	7.3 (6.4–8.3)	Ns
IL-6 < 1.6 pg/mL, *n* = 41	6.8 (5.9–7.7)	
IL-6 ≥ 1.6 pg/mL; *n* = 39	7.0 (6.1–8.0)	Ns
Haptoglobin < 1.42 *µ*g/mL; *n* = 39	6.1 (5.3–6.9)	
Haptoglobin ≥ 1.42 *µ*g/mL, *n* = 41	7.8 (6.9–8.8)	*P* < 0.01

**Table 4:** The univariate correlations of study parameters in the whole group of subjects.

	Zonulin	TNF-*α*	sTNFR1	sTNFR2	IL-6
Age	*R* = 0.43^∧^	*R* = 0.21	*R* = 0.25*	*R* = 0.27*	*R* = 0.25*
Body mass	*R* = 0.34**	*R* = 0.29*	*R* = 0.6^∧^	*R* = 0.3*	*R* = 0.51^∧^
BMI	*R* = 0.41^∧^	*R* = 0.30*	*R* = 0.67^∧^	*R* = 0.35**	*R* = 0.58^∧^
Fat mass	*R* = 0.42^∧^	*R* = 0.29*	*R* = 0.61^∧^	*R* = 0.33**	*R* = 0.57^∧^
Fat percentage	*R* = 0.40^∧^	*R* = 0.22	*R* = 0.48^∧^	*R* = 0.29*	*R* = −0.52^∧^

Diet					
Total energy intake	*R* = 0.27*	*R* = 0.04	*R* = 0.30**	*R* = 0.27*	*R* = 0.31**
Fat	*R* = 0.15	*R* = 0.16	*R* = 0.15	*R* = 0.04	*R* = 0.29*
Carbohydrates	*R* = −0.01	*R* = −0.15	*R* = −0.02	*R* = 0.04	*R* = −0.19
Protein	*R* = −0.23*	*R* = 0.05	*R* = −0.02	*R* = −0.12	*R* = −0.11
Fiber	*R* = 0.08	*R* = 0.06	*R* = 0.14	*R* = 0.07	*R* = −0.01

Biochemical parameters		
Glucose	*R* = 0.18*	*R* = 0.01	*R* = 0.08	*R* = 0.11	*R* = 0.05
Insulin	*R* = 0.12	*R* = 0.13	*R* = 0.26*	*R* = 0.27*	*R* = 0.41^∧^
HOMA-IR	*R* = 0.15	*R* = 0.17	*R* = 0.25*	*R* = 0.26*	*R* = 0.37**
HgbA1c	*R* = 0.08	*R* = 0.12	*R* = 0.01	*R* = 0.07	*R* = 0.20
Total cholesterol	*R* = 0.09	*R* = 0.05	*R* = −0.06	*R* = −0.12	*R* = −0.09
HDL-cholesterol	*R* = −0.07	*R* = −0.32**	*R* = −0.33**	*R* = −0.21	*R* = −0.38**
LDL-cholesterol	*R* = 0.21	*R* = 0.09	*R* = 0.01	*R* = −0.09	*R* = 0.02
Triglycerides	*R* = 0.19	*R* = 0.12	*R* = 0.22	*R* = 0.13	*R* = 0.28*
TNF-*α*	*R* = −0.01	—	*R* = 0.25*	*R* = 0.13	*R* = 0.37**
sTNFR1	*R* = 0.34**	*R* = 0.25*	—	*R* = 0.61^*∧*^	*R* = 0.34**
sTNFR2	*R* = 0.16	*R* = 0.13	*R* = 0.61^*∧*^	—	*R* = 0.30**
IL-6	*R* = 0.04	*R* = 0.33**	*R* = 0.37**	*R* = 0.30**	—

Gut microbiota					
Total bacterial count	*R* = 0.26*	*R* = −0.06	*R* = 0.33**	*R* = 0.18	*R* = 0.08
*Bacteroides* count	*R* = 0.08	*R* = 0.11	*R* = 0.17	*R* = 0.10	*R* = 0.11
*Firmicutes* count	*R* = 0.04	*R* = −0.22	*R* = −0.07	*R* = −0.11	*R* = −0.02
Percentage of *Bacteroides *	*R* = −0.02	*R* = 0.10	*R* = 0.04	*R* = 0.01	*R* = 0.06
Percentage of *Firmicutes *	*R* = −0.05	*R* = −0.18	*R* = −0.19	*R* = −0.21	*R* = −0.08
*Firmicutes/Bacteroides *index	*R* = 0.04	*R* = 0.20	*R* = 0.16	*R* = 0.09	*R* = 0.09

*P < 0.05; **P < 0.01; ^*∧*^ *P* < 0.001.
